# Updated Stroke Gene Panels: Rapid evolution of knowledge on monogenic causes of stroke

**DOI:** 10.1038/s41431-022-01207-6

**Published:** 2022-10-17

**Authors:** Andreea Ilinca, Andreas Puschmann, Jukka Putaala, Frank Erik de Leeuw, John Cole, Stephen Kittner, Ulf Kristoffersson, Arne G. Lindgren

**Affiliations:** 1 Department of Neurology, Skåne University Hospital; Department of Clinical Sciences Lund, Neurology, Lund University, Lund, Sweden; 2 Department of Neurology, Helsinki University Hospital, Helsinki, Finland; 3 Radboud University Medical Center, Department of Neurology; Donders Center for Medical Neuroscience, Nijmegen, The Netherlands; 4 Department of Neurology, Veterans Affairs Maryland Health Care System, University of Maryland School of Medicine, Baltimore, MA USA; 5 Department of Neurology, University of Maryland School of Medicine, Baltimore, MA USA; 6 Division of Clinical Genetics, Laboratory Medicine, Lund University, Lund, Sweden

**Keywords:** Stroke, Genetics research

## Abstract

This article updates our previous Stroke Gene Panels (SGP) from 2017. Online Mendelian Inheritance in Man and PubMed were searched. We divided detected genes into two SGP groups, SGP1: genes reported in at least one person with stroke and associated with one or more clinical subgroups: large artery atherosclerotic, large artery non-atherosclerotic (tortuosity, dolichoectasia, aneurysm, non-atherosclerotic dissection or occlusion), cerebral small vessel diseases, cardio-embolic (arrhythmia, heart defect, cardiomyopathy), coagulation dysfunctions (venous thrombosis, arterial thrombosis, bleeding tendency), intracerebral hemorrhage, vascular malformations (cavernoma, arteriovenous malformations) and metabolism disorders; and SGP2: genes related to diseases that may predispose to stroke. We identified 168 SGP1 genes, 70 of these were validated for clinical practice. We also detected 72 SGP2 genes. Nine genes were removed because of conflicting evidence. The number of genes increased from 168 to 240 during 4.5-years, reflecting a dynamic evolution and the need for regular updates for research and clinical use.

## Introduction

Monogenic conditions have an important contribution to stroke risk [1, 2] but they have been difficult to diagnose because of still incomplete knowledge on how monogenic mechanisms are related to disease, relatively expensive diagnostic methods, and because of the heterogenous and multifactorial nature of stroke. Many different monogenic conditions can cause or predispose for stroke [3]. The introduction of massively parallel sequencing methods such as whole exome and whole genome sequencing (WES, WGS) has led to the detection of more and more gene-disease associations [4, 5]. Likewise, WES and WGS have increasingly been used in clinical practice in the workup of patients with stroke where familial aggregation of stroke, the absence of classical risk factors, or young age suggest a high potential for discovering monogenic causes [6, 7]. For these purposes, a panel listing all known stroke genes is a valuable tool in research and clinical management.

The present article updates our previous comprehensive Stroke Gene Panel (SGP) publication [3] which was based on a literature search from August 2017. By using the same systematic methods to compile a new SGP 4.5-years later, we aimed to create an updated panel with all stroke-genes known to date. We also aimed to investigate how fast knowledge develops regarding the level of evidence of monogenic conditions related to stroke and cerebrovascular disease.

## Methods

### Systematic search

For the present SGP update, we used identical methods to identify genes as in our previously published SGP [3]. The systematic searches in Online Mendelian Inheritance in Man (OMIM) [8] and PubMed databases were conducted until February 2022. Genes reported to be associated with stroke were identified by using combinations of search terms: (stroke), (cerebrovascular), (cerebral OR intracerebral OR intracranial OR brain OR encephalic) AND (infarct OR infarction OR ischemia OR ischaemia), (ischemic OR ischaemic) AND (event OR stroke), (transitory OR transient) AND (event OR ischemic OR ischaemic), or (intracranial OR cerebral OR intracerebral OR encephalic OR brain) AND (haemorrhage OR haemorrhage OR bleeding OR hematoma).

### Association with stroke (SGP1) or stroke-predisposing condition (SGP2)

**Fig. 1:**
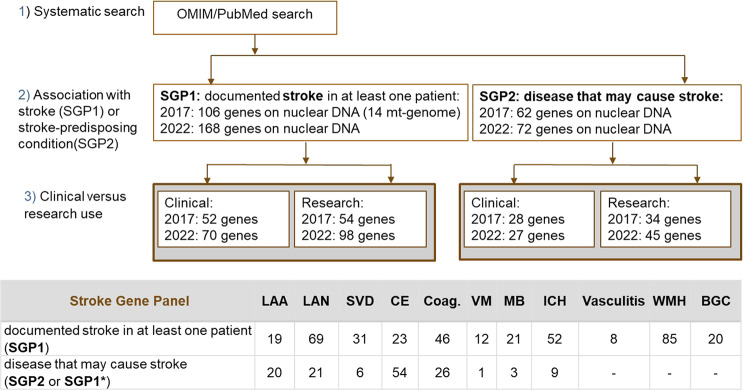
Systematic identification of genes related to monogenic (ischemic and/or hemorrhagic) stroke and a summary of the number of genes corresponding to each stroke subtype. OMIM Online Mendelian Inheritance in Man, SGP1 Stroke Gene Panel 1, SGP2 Stroke Gene Panel 2, LAA large artery atherosclerosis, LAN large artery non-atherosclerotic, SVD small vessel disease, CE cardioembolic, Coag coagulation, MB metabolic, ICH hemorrhage, VM vascular malformation, WMH white matter hyperintensities, BGC basal ganglia calcifications; * the clinical subcategory marked with “*” corresponds to SGP2 while the gene was mentioned in SGP1 because other clinical subcategories fulfill the definition of SGP1. Other characteristics as the presence of vasculitis, basal ganglia calcifications and white matter hyperintensities were evaluated only for the genes in SGP1. While our 2017 gene panel listed 14 genes in the mitochondrial DNA, we have not included genes on the mitochondrial genome in the 2022 update because determining the disease association for single genes on the mitochondrial DNA is very difficult. We recommend including the whole mitochondrial genome in genetic analyses of stroke patients and then evaluate the literature and database information on particular variants.

Cerebrovascular conditions for whom the molecular basis of the disorder is known (OMIM phenomap key 3,4) [8] are shown in the updated SGPs. Genes on nuclear DNA containing at least one mutation where a causative role has been shown or postulated were included in the panels. We again compiled two subpanels SGP1 and SGP2. If the gene was reported to cause at least one well-documented stroke case (PubMed), it was included in SGP1. The OMIM search also retrieved genes for diseases that predispose to stroke but where no patients with stroke were reported in the literature; these genes were included in SGP2 when diseases caused by mutations in the gene were documented in at least one patient in publications from PubMed (Fig. 1). In cases where one gene was associated with several clinical phenotypes of stroke where the level of evidence differed, genes already included in SGP1 were not included again in SGP2.

Each gene included in the SGPs was associated with one or more stroke-subtypes whenever this information could be found in published case reports. We amended the Causative Classification of Stroke (CCS)/Trial of Org 10172 in Acute Stroke Treatment classifications (TOAST) [9, 10] to better reflect pathobiology and thus—presumably—genetics. The following eight major stroke/cerebrovascular disease subtypes were used [3] (# indicates subtypes summarized as “other causes” in CCS) as well a separate category for intracerebral bleeding.

1. large artery atherosclerotic (*LAA*)
2. large artery nonatherosclerotic (*LAN*)#
3. small vessel disease (*SVD*)
4. cardio-embolic (*CE*- arrhythmia, morphologic cardiac defect, cardiomyopathy)
5. coagulation defects (*coag*.- arterial thrombosis, venous thrombosis, bleeding)#
6. vascular malformations (*VM*)#
7. metabolic disorders (*MB*)#
8. intracerebral bleeding (*ICH*)#

Additional characteristics of the main 8 categories of stroke in SGP1, regarding LAN-vasculitis, basal ganglia calcifications and white matter hyperintensities were also considered. We did not include age at stroke onset for each gene because the phenotypic variability regarding age at first stroke onset can be substantial.

### Clinical versus research use

**Fig. 2:**
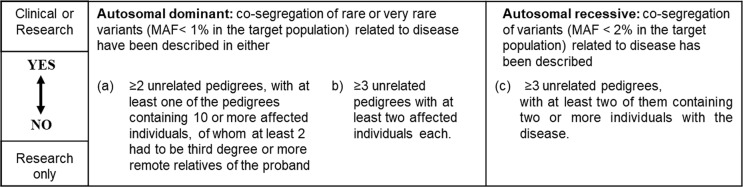
Genes for research screening or for both clinic and research screening. MAF minimum allele frequency.

The genes included in SGP1 and SGP2 are not equally well documented to be disease-associated in the literature. We thus further evaluated if the evidence for each gene was sufficient to consider this gene for clinical genetic testing (marked “C”), or if the gene only should be considered in research (“R”). We define clinically useful stroke genes based on clinical and co-segregation criteria, in a way that would correspond to at least a moderate level of supportive evidence in established recommendations for clinical validity of gene-disease associations [11]. We based this evaluation on the number of published families where the clinical phenotype co-segregated with the gene variant (Fig. 2) and used the following criteria for considering genes suitable for clinical screening:

- For autosomal dominant inheritance: genes where co-segregation of rare or very rare variants (minor allele frequency below 1% in the target population) have been related to disease in either:

- two or more unrelated pedigrees, with at least one of the pedigrees containing 10 or more affected individuals, of whom at least 2 had to be third degree or more remote relatives of the proband, or
- three or more unrelated smaller pedigrees with at least two affected individuals each.

- For autosomal recessive inheritance: genes where co-segregation of variants (with a minor allele frequency below 2% in the target population) have been related to disease in:

- at least three unrelated pedigrees, with at least two of them containing two or more individuals with the disease.

The pathogenicity of variants identified in these genes need then to be individually evaluated—as suggested in existing guidelines [12] by using existing databases containing regularly updated information on genetic variants.

Genes where conflicting information on disease-causing effect was reported were considered for research purpose only. The stroke-genes were identified through the same methods in 2017 and 2022, allowing a comparison between the number of stroke-genes.

## Results

**Fig. 3:**
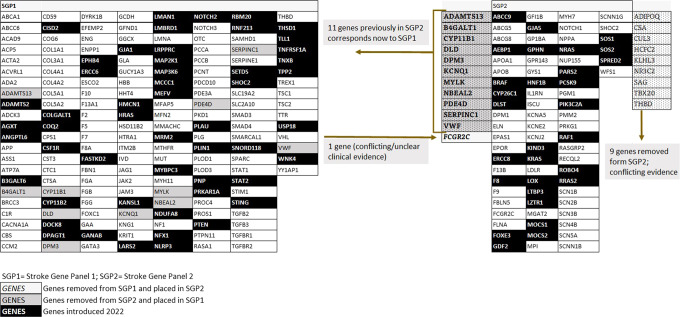
Genes newly introduced to SGP1 and SGP2. SGP1 Stroke Gene Panel 1, SGP2 Stroke Gene Panel 2. The genes in dark rubrics are newly identified and the ones in light rubrics are replaced from one SGP to the other SGP.

In total, we identified 168 **SGP1** genes and 72 SGP2 genes. Compared with our earlier publication [3], 63 new stroke-genes were included in SGP1 (Supplemental Material). Among these, 52 were newly reported genes and 11 were genes previously included in SGP2 but now fulfilling the criteria for inclusion in SGP1 (Fig. 3). These 63 newly included genes in SGP1 were associated with the following phenotypes: 5 (LAA), 21 (LAN), 7 (SVD), 8 (CE), 10 (coagulopathies), 3 (vascular malformations), 11 (metabolic phenotype) and 16 (ICH) (Fig. 1). Re-evaluation showed that for three stroke-genes previously considered suitable for clinical screening, the relevance for stroke had become too inconsistent, and therefore they are now only recommended for research (*CACNA1A, MYLK, MFN2*), whereas the evidence for 10 other stroke-genes was strengthened and now fulfill our criteria for clinical testing. Twenty-eight new stroke-genes fulfilling the inclusion criteria for SPG2 (Supplemental Material) were identified (Fig. 3). One gene from SGP1 was now placed in SGP2 because of conflicting clinical evidence (*FCGR2C*) and nine genes from SGP2 were removed from SGP2 because conflicting evidence has emerged *(ADIPOQ, CSA, CUL3, HCFC2, KLHL3, NR3C2, SAG, TBX20, THBD)*.

## Discussion

As has been suggested for other disorders, the diagnostic yield of WES and WGS tests of stroke patients may increase if data are regularly re-evaluated and the gene panels are updated [6, 13] and used in combination with recent detailed information on the phenotype.

During the last 4.5 years additional monogenic causes for stroke have been identified at a rapid pace. By using the same search algorithm as in 2017, SGP1 increased by 60% of genes, and 30% more SGP1 stroke-genes now fulfill the criteria for genetic testing in clinical practice. Also, the number of genes in SGP2 increased. However, evolving knowledge also revealed that nine genes (5,4%), that were associated with monogenic stroke based on the evidence available in 2017, now no longer fulfill criteria for our SGPs (Fig. 3).

Our SGP contain clinical information on stroke subtypes. Besides the three established standardized TOAST/CCS subtypes of ischemic stroke [9, 10], we again used five additional subtypes (marked with a # sign in the Methods section) to delineate those “other causes” that frequently occur among the monogenic forms of stroke and that may represent distinct molecular and pathogenic mechanisms. Furthermore, in SGP1 we now also systematically included information on three other associated characteristics (large artery vasculitis, white matter hyperintensities, bilateral basal ganglia calcifications) to facilitate the correct interpretation of a genetic variant found in a stroke patient or family. Given the large spectrum of possible stroke mechanisms, an accurate matching between the stroke phenotype in the patient/family under investigation with the phenotype described in patients with pathogenic variants in the same gene increases the likelihood that the identified variant is truly disease-causing. As WES and WGS examine all genes simultaneously, false-positive “chance” findings are possible. Misinterpretation of such findings can be minimized when only considering genes for which the known clinical phenotype corresponds to the one in the patient under investigation [6, 14].

We compiled our panel by using a systematic and highly replicable approach that allowed us to accurately compare the number of stroke genes in 2017 with 2022. We are aware that this approach has missed genes that fulfill the criteria for SGP1 or SGP2 but that were not retrieved by our methodology. This includes some of the genes for moyamoya phenomenon, other vascular malformations, abnormalities of coagulation including CBL [15], DIAPH1 [16, 17], CHD4, CNOT3, and SETD5 [16]. This inherent difficulty in compiling gene panels is well known. Furthermore, too extensive panels may increase the yield of variants that are not relevant to the disease phenotype [18, 19].

While SGPs offer an evidence-based list of stroke-genes with specified level of evidence as clinical or research only, they do not offer specific guidance for variant interpretation. Complementary resources and available expert knowledge are needed to support clinicians in interpreting the variant pathogenicity [12] and the level of actionability [20].

## Supplementary information

Stroke Gene Panel 1

Stroke Gene Panel 2

Supplemental Material

## Data Availability

Data generated during this study can be found within the published article and its supplementary files.
