# Supplementary material for: Updated Stroke Gene Panels: Rapid evolution of knowledge on monogenic causes of stroke
Source: Eur J Hum Genet. 2022 Oct 17;31(2):239–42. doi: 10.1038/s41431-022-01207-6 (PMC9905069; doi:10.1038/s41431-022-01207-6)
Supplement: Supplementary file 3 — Supplemental Material [file 41431_2022_1207_MOESM3_ESM.docx]

***Supplemental Material***

SGP1 and SGP2 (two excel files)

SGP1: Gene variations related to stroke documented in at least one patient.

SGP2: Gene variations related to an intermediary phenotype for stroke documented in at least one patient (publications/PubMed).
